# Influence of Age on Cardiorespiratory Kinetics During Sinusoidal Walking in Humans

**DOI:** 10.3389/fphys.2018.01191

**Published:** 2018-08-24

**Authors:** Naoyuki Ebine, Alharbi Ahad-Abdulkarim-D, Yuki Miyake, Tatsuya Hojo, Daijiro Abe, Masahiro Horiuchi, Yoshiyuki Fukuoka

**Affiliations:** ^1^Faculty of Health and Sports Science, Doshisha University, Kyoto, Japan; ^2^Center for Health and Sports Science, Kyushu Sangyo University, Fukuoka, Japan; ^3^Division of Human Environmental Science, Mount Fuji Research Institute, Fujiyoshida, Japan

**Keywords:** age, ventilation, heart rate, energy cost, sinusoidal walking

## Abstract

We sought to determine the influence of age on cardiorespiratory kinetics during sinusoidal walking in two groups: 13 healthy young subjects (YG; 7 men and 6 women, age 21 ± 2 years) and 15 healthy elderly subjects (ELD; 9 men and 6 women, age 67 ± 5 years). A treadmill’s speed was sinusoidally changed between 3 and 6 km h^-1^ in the YG and between 3 and 5 km h^-1^ in the ELD during periods of 1, 2, 5, and 10 min, and in a stepwise manner. We compared the groups’ heart rate (HR), ventilation ($\dot{V}$_E_), and gas exchange (CO_2_ output ($\dot{V}$CO_2_) and O_2_ uptake ($\dot{V}$O_2_)) responses. We determined the phase shift (*PS*) and the normalized amplitude (*Amp*) ratio of these kinetics in relation to the sinusoidal change in walking speed in response to the magnitude from the maximum to minimum speeds as revealed by a Fourier analysis in all cardiorespiratory variables. Both the *Amp* ratio and *PS* in the $\dot{V}$_E_, $\dot{V}$CO_2_, and $\dot{V}$O_2_ responses were very similar between the ELD and YG, and being independent of the periods of sinusoidal oscillations. In marked contrast, the *PS* of the HR kinetics was significantly slowed in the ELD compared to the YG. The *Amp* ratio of HR was not related to the covariance variation of HR (CVHR) at standing rest in the ELD. The HR kinetics during sinusoidal walking may not be attributable to parasympathetic nerve activity into the heart in the ELD. The slope of the *Amp* of $\dot{V}$_E_ related to the *Amp* of $\dot{V}$CO_2_ ($\dot{V}$_E_/$\dot{V}$CO_2_ slope) was steeper in the ELD (0.0258) compared to the YG (0.0132), suggesting that exercise hyperpnea could be greatly induced during walking in the ELD. These findings suggest that aging influences the alterations of autonomic nervous system-dependent slower HR kinetics and exercise hyperpnea during walking in the ELD.

## Introduction

The cardiorespiratory readjustments that take place when a human body changes from a rest state to exercise become slower with advancing age (Paterson et al., 1987; Babcock et al., 1992, 1994; Bell et al., 1999). Cunningham et al. (1992) compared the gas exchange of oxygen uptake ($\dot{V}$O_2_), carbon dioxide output ($\dot{V}$CO_2_), ventilation ($\dot{V}$_E_), and heart rate (HR) kinetics to the sinusoidal work rate in younger and older women, and they found that the HR, $\dot{V}$O_2_, and $\dot{V}$CO_2_ kinetics became relatively sluggish in older women. In our recent observation, the lesser changes in $\dot{V}$O_2_, $\dot{V}$CO_2_, and $\dot{V}$_E_ kinetics against sinusoidal change in walking speed were remarkably different from a deceleration in HR kinetics under moderate hypoxia compared to normoxia (Ebine et al., 2018).

A sinusoidal work load protocol has been used to more precisely define cardiorespiratory kinetic characteristics (Miyamoto et al., 1983; Fukuoka et al., 1995). Dynamic physiological characteristics can be estimated by the phase shift (*PS*) as the “time lag” and the amplitude (*Amp*) as the “responsiveness” of cardiorespiratory variables in response to sinusoidal exercise (Casaburi et al., 1977; Bakker et al., 1980; Haouzi et al., 1992, 2004; Fukuoka et al., 2002). Sinusoidal work loading has an advantage in that an alteration of the midpoint work load (between the minimum and maximum work load) would modify the variation of balance between the sympathetic and parasympathetic outflows during exercise. We demonstrated that the HR kinetics in response to moderate sinusoidal work (i.e., cycling) are related to advancing age (Fukuoka et al., 2002). Sone et al. (1997) observed that the increased HR below 100 beats min^-1^ would seem to reflect the weakening in the contribution of the parasympathetic system to HR regelation during sinusoidal cycling in young subjects. Similar mechanisms have also been suggested by Rowell (1993). The cardiac parasympathetic tone was drastically reduced (by >110 beats min^-1^) in the HR response during an incremental work test (Yamamoto et al., 1991). Thus, these previous information suggested that HR less 100–110 beats min^-1^ during walking in a sinusoidal manner can reveal the parasympathetic nerve activity to HR regulation during walking. However, Itoh et al. (2007) showed that aging depresses the parasympathetic nerve activity using the Fourier heart rate variability analysis even at resting condition, suggesting that predominant control of the HR in elderly individuals would be done by sympathetic nerve activity. We thus hypothesized that a locomotion-induced withdrawal of parasympathetic nerve activity in the elderly individuals could adjust the slower HR kinetics during sinusoidal walking.

During walking at a given constant speed, the energy cost of transport during walking per unit distance (CoT) in the elderly is inversely proportional to the slower preferred walking speed (Schrack et al., 2016). When an elderly individual walks at a fixed walking speed, the walking speed is not necessarily optimized (Selinger et al., 2015), resulting in relatively greater energy cost. This could also be attributable to an additional cardiorespiratory cost (Horiuchi et al., 2017). Our second hypothesis was that the CoT values could be greater in elderly subjects compared to young subjects.

Moreover, according to the cardiodynamic hypothesis, $\dot{V}$_E_ kinetics in the transient phase are mediated by the pulmonary vascular CO_2_ flow to the lungs (Whipp, 1994). Thus, a potential increase in the $\dot{V}$_E_ in the elderly individuals is related to the $\dot{V}$CO_2_ value (i.e., the slope of $\dot{V}$_E_–$\dot{V}$CO_2_ linkage), by which a greater dead space in bronchial pathways could develop due to aging (Mucci et al., 1998). Consequently, such a potential increase in the $\dot{V}$_E_ without an effective gas exchange in the pulmonary area impedes the gas exchange efficiency, which leads to the increased gas exchange work during walking. Here, our third hypothesis was established that the steeper slope of the $\dot{V}$_E_–$\dot{V}$CO_2_ linkage in the elderly individuals compared to young individuals would be exaggerated when the walking speed is sinusoidally changed.

To test these three hypotheses, we investigated whether the cardiorespiratory responses showed different kinetics between young adult and elderly subjects against sinusoidal walking speed changes at an equivalent metabolic demand.

## Materials and Methods

### Subjects

Thirteen healthy young adults (the YG; 7 males and 6 females; mean ± standard deviation (SD) age: 21 ± 2 years, height: 165 ± 7 cm, weight: 58 ± 9 kg) and 15 healthy elderly adults (the ELD; 9 males and 6 females; age: 67 ± 5 years, height: 160 ± 7 cm, weight: 59 ± 9 kg) participated without medication histories to affect cardiovascular function. All elderly females were post-menopausal women and all measurements in young females were carried out at follicular phase in menstrual cycle. Although physical activity in elderly individuals could not be measured, they have not performed regular sports activity for the past 3 years. They were fully informed of possible risks and discomforts associated with the experiments before giving their written informed consent to participate in the study, which was approved by the ethics committees of the Institutional Review Board of Doshisha University (No. 1045).

### Exercise Protocol

Before walking, all of the subjects stood for 4 min to achieve a stable HR at standing rest on the treadmill. Before the sinusoidal change in walking speed, we carried out a stepwise protocol (constant) at speeds of 3 km h^-1^ for 5 min in both groups; then, for the next 3 min, 6 km h^-1^ was applied in the YG subjects and 5 km h^-1^ was applied in the ELD subjects (Fukuoka et al., 2017; Ebine et al., 2018). The atmosphere in the environmental chamber was set at 23–25°C with a relative humidity of 50%.

Following a 4-min warm-up consisting of walking at a constant speed at the midpoint of the sinusoid change (i.e., 4.5 km h^-1^ in the YG, 4 km h^-1^ in the ELD), the treadmill speed was changed with a sinusoidal pattern from 3 to 6 km h^-1^ in the YG and from 3 to 5 km h^-1^ in the ELD at periods of 10, 5, 2, and 1 min. Following a 4-min warm-up at a constant speed at the midpoint between maximum and minimum walking speed, the sinusoidal loading was repeated for six cycles in 1-min periods and continued for three cycles in 2-min periods. On another day, following a 5-min warm-up at a constant speed of 4.5 km h^-1^ in the YG and 4 km h^-1^ in the ELD, another sinusoidal walking load was repeated for three cycles in 5-min periods and continued for two cycles in 10-min periods. The subjects walked on the treadmill at a freely chosen pace.

### Measurements

A mass-flow sensor (type AB, Minato Medical Sciences, Osaka, Japan) was fit to the expiratory port of the valve to continuously record the subject’s expiratory airflow, which was calibrated before each measurement with a 3-L syringe at three different flow rates. We calculated the tidal volume (VT) and $\dot{V}$_E_ by integrating the flow tracings recorded at the subject’s mouth. We confirmed that the sensitivity of the hot-wire anemometer did not alter with changes in gas concentrations over the range of physiological flow variations. Expiratory PO_2_ and PCO_2_ were determined by mass spectrometry (Arco-2000, Arco System, Chiba, Japan) from a sample drawn continuously from the inside of the mouthpiece at 1 ml s^-1^; the loss of volume, however, was neglected in our calculations. Three reference gases of known concentrations (O_2_ 15.04%, CO_2_ 2.92%, and N_2_ 82.04%; O_2_ 11.93%, CO_2_ 6.96%, and N_2_ 81.11%) and room air (O_2_ 20.93%, CO_2_ 0.03%, Ar 0.94%, and N_2_ 78.10%) were used to calibrate the mass spectrometer.

The volumes, flows, PCO_2_ and PO_2_ at the mouth were recorded in real time with a 50-Hz sampling frequency using a computerized on-line breath-by-breath system (AE-280, Minato Medical Sciences, Osaka, Japan) from time-aligned gas volume and concentration signals. Breath-by-breath $\dot{V}$_E_ (BTPS), $\dot{V}$O_2_ (STPD), and $\dot{V}$CO_2_ (STPD) were determined. An electrocardiogram (ECG) was collected through a bioamplifier (AB 621G, Nihon Kohden, Tokyo, Japan). Heart rate (HR) was measured by beat-by-beat counting from the *R* spike of the ECG. The signals from the treadmill were fed into a data acquisition system (PowerLab system, A/D Instruments, Castle Hill, NSW, Australia) and temporally aligned to the ventilatory and ECG data.

The signal controlling the speed of the motor driving the treadmill (modified TMS 2200, Nihon Kohden) was delivered by a microcomputer through a digital–analog converter.

### Data Analysis

During the constant-speed walking before the sinusoidal speed change, the breath-by-breath $\dot{V}$O_2_ (ml kg^-1^ min^-1^) and the $\dot{V}$CO_2_ (ml kg^-1^ min^-1^) at three constant walking speeds (3, 4, and 5 km h^-1^ in the ELD; 3, 4.5, and 6 km h^-1^ in the YG) on the treadmill were also continuously measured. A single sample of the average $\dot{V}$O_2_ and $\dot{V}$CO_2_ for the final 1 min at each gait speed was used to calculate the energy expenditure (EE: J kg^-1^ min^-1^) using the following equation (Brouwer, 1957; Masschelein et al., 2012).

(1) $$\mathrm{EE}(J{\mathrm{kg}}^{-1}\;\min^{-1})\;=\;4.186\times [(3.869\times \dot{V}O_{2})+(1.195\times \dot{V}{\mathrm{CO}}_{2})]$$

The EE was divided by speed (*v*: m min^-1^) to obtain the energy cost of walking per unit distance (CoT; J kg^-1^ m^-1^) during walking at a given constant speed as follows.

(2) $$\mathrm{CoT}\;=\;\mathrm{EE}\cdot v^{-1}$$

The CoT-*v* relationship can be mathematically described by the following equation (Wall-Scheffler and Myers, 2013; Abe et al., 2017).

(3) $$\mathrm{CoT}(v)\;=\;av^{2}\;+\;bv\;+c$$

where the constants a, b, and c are determined by the least squares regressions with data obtained from three walking speeds (Ortega et al., 2014). For Comparing the CoT values between the YG and ELD, the CoT values at gait speeds of 4 and 5 km h^-1^ were calculated by and interpolation using the *eq.* 3 in the YG.

We performed a Fourier analysis to analyze all of the sinusoidal data, as described (Wigertz, 1971; Haouzi et al., 1992, 2004; Fukuoka et al., 1997, 2002). The repeated cardiorespiratory responses to sinusoidal walking speed were overlapped in correspondence with the cycle period, and we obtained the mean cardiorespiratory data at each respective cycle. The variation in the speed of the treadmill was regarded as the input function. The *Amp* (i.e., mean to peak) and the *PS* of the fundamental component (the same frequency as the input function) of the $\dot{V}$_E_, $\dot{V}$O_2_, $\dot{V}$CO_2_, and HR responses were computed as follows:

(4) $$Amp\;=\;\sqrt{Re^{2}\;+\;Im^{2}}$$

and

(5) $$PS\;=\;\tan^{-1}\;\left(\frac{Re}{Im}\right)$$

where the *Re* and *Im* are the real and imaginary components; these were calculated as follows. The larger the *PS*, the slower the response. The larger the *Amp*, the higher the responsiveness.

(6) $$Re\;=\;\frac{2}{NT}\sum_{t=0}^{\mathrm{NT}}[(x(t)-Mx)\;\cos(2\pi ft)]$$

and

(7) $$Im\;=\;\frac{2}{NT}\sum_{t=0}^{\mathrm{NT}}\left[(x(t)-Mx)\;\sin(2\pi ft)\right]$$

where *x*(*t*) is the response value at time *t* (in s), *Mx* is the mean value of *x* for an integer number of cycles (*N*), *T* is the period of the input signal (in s) and *f* (=1/*T*) is its frequency in cycles per second. Because we set different walking speeds for the ELD and YG groups, we calculated the *Amp* ratio for each parameter to the sinusoidal speed variation normalized by dividing by magnitude of each parameter from 3 to 6 km h^-1^ during the constant walking (Fukuoka et al., 1995).

The R-R intervals during sinusoidal work were calculated beat-by-beat by the computer, and 1-s interval HR data were measured from the calculated R-R intervals (R-R) and converted as HR (60/R-R). We then determined the covariance of the variance of HR relative to the mean of the HR data (i.e., the CVHR) at rest as an indicator of parasympathetic nerve activity (Eckberg, 1983; Fukuoka et al., 2002).

### Statistical Analyses

All values are presented as mean ± SD. The significance of differences in each variable ($\dot{V}$_E_, $\dot{V}$O_2_, $\dot{V}$CO_2_, and HR) was determined by a two-way repeated measures analysis of variance (ANOVA) in the comparison of age groups (YG and ELD) × oscillation frequency period (*T*; 1–10 min) and the *Newman–Keuls* test. The CoT values of the YG and ELD were also compared by a two-way ANOVA in the comparison of age groups (YG and ELD) × waking speeds (4 and 5 km h^-1^) and the *Newman–Keuls* test. The value of *H* (after correction for similar values) and the corresponding *p*-values are given in the text for each variable. We compared the regression coefficients of the independent variables of $\dot{V}$_E_ between the YG and ELD groups. The level of significance was set at *p* < 0.05.

## Results

### Impact of Aging on the Energy Expenditure During Constant Walking

A significantly greater CoT was found at 3 km h^-1^ in the ELD compared to the YG (4.947 ± 0.913 vs. 4.051 ± 0.525 J kg^-1^ m^-1^ for the ELD and YG, respectively, *p* < 0.01). At 4 and 5 km h^-1^, the CoT was also significantly greater in the ELD than that of the YG (*p* < 0.01) (**Table 1**).

**Table 1:** Mean values of the energy cost of walking per unit distance (CoT) between the young and elderly groups at three different walking speeds.

	*Speed*		3 km h^-1^		4 km h^-1^		5 km h^-1^	
Young	Mean		4.051^∗∗^		3.874^∗∗^		3.734^∗∗^	J kg^-1^ m^-1^
	SD	±	0.525	±	0.446	±	0.387	
	*Speed*		3 kmh^-1^		4 kmh^-1^		5 kmh^-1^	
Elderly	Mean		4.947		4.485		4.209	J kg^-1^ m^-1^
	SD	±	0.913	±	0.733	±	0.690	


*^∗∗^p < 0.01 vs. elderly. Data are shown by mean ± SD.*

**FIGURE 1:**
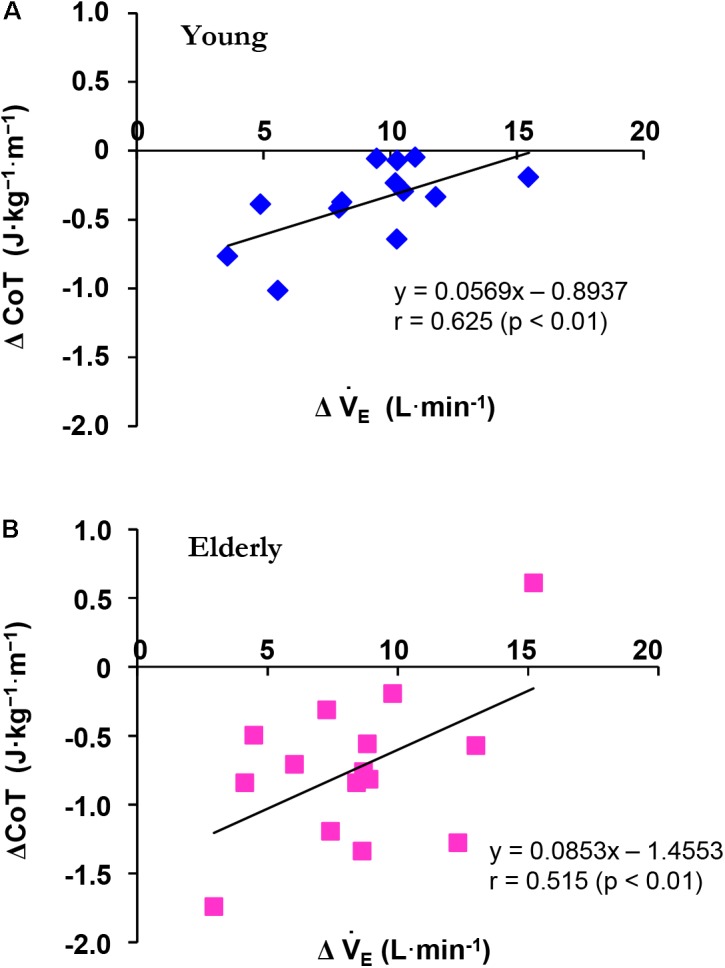
Relationship between the ΔCoT and Δ$\dot{V}$_E_ during constant walking in the young (YG, **A**) and elderly (ELD, **B**) subjects. Note that the ΔCoT was significantly related to Δ$\dot{V}$_E_ in both the YG (*blue*) and ELD (*red*) groups. The regression line between the ΔCoT and Δ$\dot{V}$_E_ was significant in both the YG (*r* = 0.625, *p* < 0.01) and ELD (*r* = 0.515, *p* < 0.01) groups.

To facilitate comparisons between the YG and the ELD, we calculated the changes in $\dot{V}$_E_ (Δ$\dot{V}$_E_) and CoT (ΔCoT) from 3 to 5 km h^-1^ in the ELD and from 3 to 6 km h^-1^ in the YG during the constant walking. There was a significant correlation between the ΔCoT and Δ$\dot{V}$_E_ from 3 to 5 km h^-1^ in the ELD (*r* = 0.515, *p* < 0.01) and from 3 to 6 km h^-1^ in the YG (*r* = 0.625, *p* < 0.01), thus revealing a relationship between ΔCoT and Δ$\dot{V}$_E_ (**Figure 1**).

### Kinetics of Cardiorespiratory Parameters During Sinusoidal Walking

**FIGURE 2:**
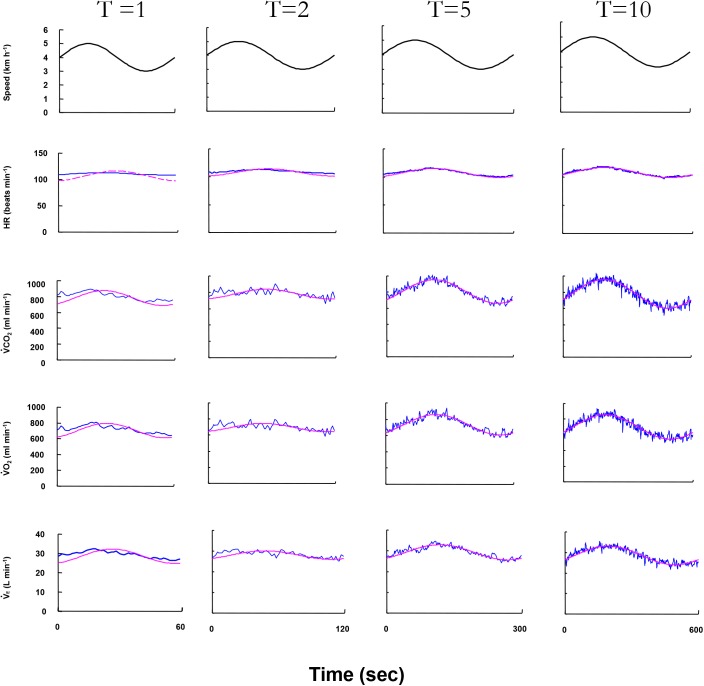
The cardiorespiratory variables of ventilation ($\dot{V}$_E_), O_2_ uptake ($\dot{V}$O_2_), CO_2_ output ($\dot{V}$CO_2_), and heart rate (HR) responses to four different treadmill speed oscillation during 1-, 2-, 5-, and 10-min periods in a representative elderly subject. *Oscillating line:* superimposed gas exchange variables data. *Smooth line:* sine-wave fundamental component of these kinetics. The HR response at the 1-min period could not be estimated by Fourier analysis.

**Figure 2** displays the superimposed cardiorespiratory kinetics obtained from a representative ELD subject and the calculated fundamental components during walking for all periods. As shown in the figure, the *Amp* and *PS* are reliable variables that could be used to estimate the fundamental component of the cardiorespiratory kinetics during walking. With prolonged periods of sinusoidal walking, larger *Amp* values and smaller *PS* values were observed as cardiorespiratory kinetics.

**FIGURE 3:**
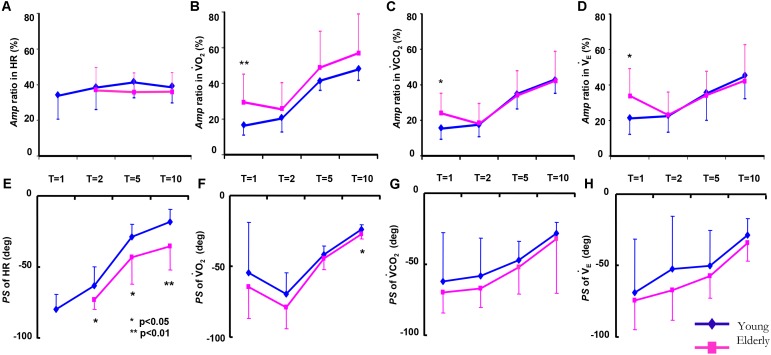
Comparison of the amplitude (*Amp*) ratio (*A* ratio for each variable between the constant and sinusoidal load intensity variation) and the phase shift (*PS*) of the HR **(A,E)**, $\dot{V}$O_2_ **(B,F)**, $\dot{V}$CO_2_ **(C,G)**, and $\dot{V}$_E_ **(D,H)**, between the elderly individuals (*red line*) and the younger individuals (*blue line*) as a function of the periods of sinusoidal changes in treadmill speed. Data are mean ± SD. ^∗^*p* < 0.05, ^∗∗^*p* < 0.01 vs. ELD.

In the HR kinetics in the 1-min period, since sinusoidal HR oscillation could not be clearly detected by Fourier analysis, the *Amp* and *PS* values of the HR could not be obtained in the ELD group. Significantly greater absolute *Amp* values of all variables at all periods were observed in the ELD compared to the YG (*p* < 0.01) because we set different walking speeds for the two groups. There were no significant differences in the *Amp* ratios of $\dot{V}$_E_, $\dot{V}$CO_2_, $\dot{V}$O_2_, or HR responses at all periods except the 1-min period (**Figures 3A–D**).

An increase in the *PS* in cardiorespiratory kinetics (slower response) occurred when the period of the oscillations of the treadmill speed decreased (**Figures 3E–H**). Of the cardiorespiratory kinetics, the *PS* of the $\dot{V}$_E_, $\dot{V}$CO_2_, and $\dot{V}$O_2_ responses tended to be smaller (faster response) but were not significantly different between the ELD and YG groups at any periods of sinusoidal walking except 10 min (*p* < 0.05). In contrast, the *PS* of the HR response was significantly larger (slower response) in the ELD compared to that of the YG (*p* < 0.05 or 0.01) at the periods of 2, 5, and 10 min.

**FIGURE 4:**
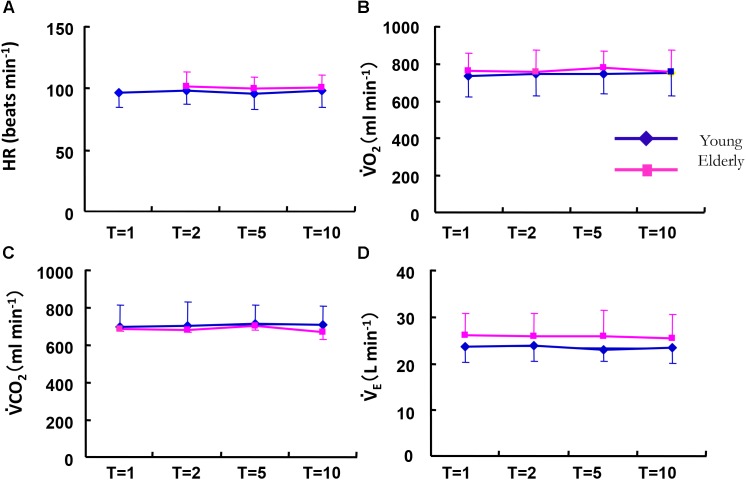
Comparison of the averaged mean values (*Mx*) of HR **(A)**, $\dot{V}$O_2_ **(B)**, $\dot{V}$CO_2_ **(C)**, and $\dot{V}$_E_ **(D)** between the elderly (*red line*) and younger individuals (*blue line*) as a function of the period of the sinusoidal changes in treadmill speed. There were no significant differences in all variables between the YG and ELD in any of the different periods. Note that the metabolic demand was similar between the YG and ELD during sinusoidal walking. Data are mean ± SD.

The absolute mean values (*Mx*) of the cardiorespiratory variables of HR, $\dot{V}$O_2_, and $\dot{V}$CO_2_ kinetics during sinusoidal walking were not significantly different between the ELD and YG groups (**Figure 4**) despite the different maximum walking speeds between the ELD and YG groups. The *Mx* of $\dot{V}$O_2_ showed a narrow range from 754 to 774 ml min^-1^ in the ELD subjects and from 731 to 748 ml min^-1^ in the YG subjects in all of the periods. The *Mx* values of $\dot{V}$CO_2_ ranged from 665 to 697 ml min^-1^ in the ELD group and from 693 to 712 ml min^-1^ in the YG. By contrast, the *Mx* of $\dot{V}$_E_ in the ELD tended to be somewhat higher than that in the YG at all periods of sinusoidal walking, without a significant difference between the YG and the ELD data. Similarly, the *Mx* values of breath frequency (B*f*) and VT during sinusoidal walking at all periods were also somewhat higher in the ELD compared to the YG without significant difference between the ELD and the YG (B*f*: 26.0 ± 2.8 vs. 24.6 ± 5.0 breaths min^-1^, VT: 0.993 ± 0.147 vs. 0.988 ± 0.201 L for the ELD and the YG). Second resultant *Mx* of end-tidal PCO_2_ (P_ET_CO_2_) was tended to be lower in the ELD than that in the YG (39.8 ± 3.2 vs. 41.8 ± 2.1 mmHg for the ELD and the YG, respectively, *p* = 0.068).

**FIGURE 5:**
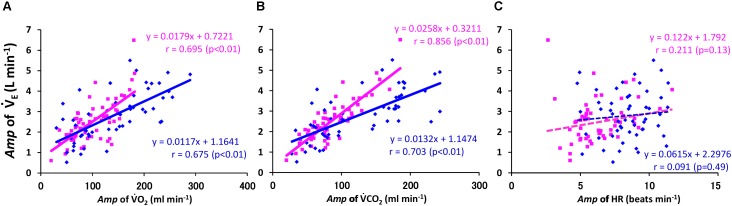
Relationship between *Amp* of the $\dot{V}$_E_ and $\dot{V}$O_2_ **(A)**, $\dot{V}$CO_2_ **(B)**, and HR **(C)**. Note that the *Amp* for the $\dot{V}$_E_ kinetics followed the *Amp* for the $\dot{V}$CO_2_ kinetics more closely. The regression line for the *Amp* of the $\dot{V}$_E_–$\dot{V}$CO_2_ relationship was steeper in the ELD than in the YG. YG; *y* = 0.0132*x* + 1.147, *r* = 0.703 (*p* < 0.01, **A**), ELD; *y* = 0.0258*x* + 0.321, *r* = 0.856 (*p* < 0.01, **B**). No significant correlation between the $\dot{V}$_E_ and the HR kinetics was found in both groups **(C)**.

When we focused the slope of the $\dot{V}$_E_–$\dot{V}$CO_2_ linkage ($\dot{V}$_E_/$\dot{V}$CO_2_), the absolute *Amp* for the $\dot{V}$_E_ response followed the *Amp* for $\dot{V}$CO_2_ (*r* = 0.856, *p* < 0.01) significantly more closely than that for $\dot{V}$O_2_ (*r* = 0.695, *p* < 0.01) or HR (*r* = 0.211, *p* = 0.107) in the ELD group among all the data of sinusoidal walking (**Figures 5A–C**). When we calculated each regression line for the *Amp* for $\dot{V}$_E_ to that for $\dot{V}$CO_2_ in the ELD and YG groups, the slope of the *Amp* of $\dot{V}$_E_ related to the *Amp* of $\dot{V}$CO_2_ (i.e., the $\dot{V}$_E_/$\dot{V}$CO_2_ slope) was two times steeper in the ELD group compared to the YG (slope, 0.0258 vs. 0.0132, respectively) (**Figure 5B**). The correlation coefficient was significantly higher in the ELD (*r* = 0.856, *p* < 0.01) compared to the YG (*r* = 0.703, *p* < 0.01).

**FIGURE 6:**
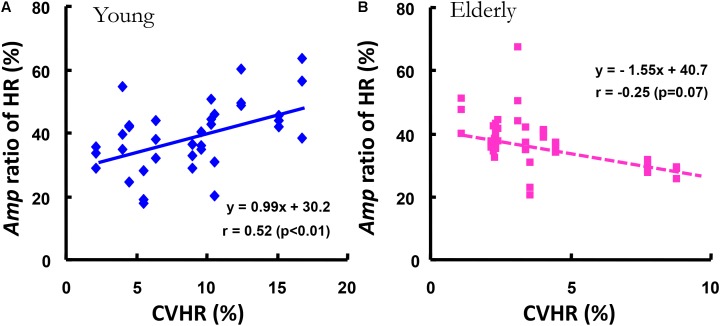
The relationship between the *Amp* ratio of the HR kinetics and the covariance of variance of HR during standing (CVHR). The *Amp* ratio of the HR kinetics was significantly related to the CVHR in the YG (*r* = 0.52, *p* < 0.01, **A**), but not in the ELD **(B)**.

In addition, a significant correlation between all *Amp* ratio of HR kinetics at the periods of 2, 5, and 10 min except 1-min and the CVHR was observed in the YG (*r* = 0.52, *p* < 0.01), but in the ELD a significant relationship was not found (*r* = -0.25, *p* = 0.068, **Figure 6**).

## Discussion

To our knowledge, this is the first study to characterize cardiorespiratory kinetics during mild exercise (i.e., walking) to determine the age-related changes in ventilatory and cardiac function. The experiment showed following results. (1) The *Amp* ratio during sinusoidal walking in HR, $\dot{V}$O_2_, $\dot{V}$CO_2_, and $\dot{V}$_E_ were not different except for the values in 1-min periods. On the other hand, the *PS* was different in HR, but not in $\dot{V}$O_2_, $\dot{V}$CO_2_, and $\dot{V}$_E_. (2) The *Amp* ratio of HR was significantly related to the covariance of the variance of HR (CVHR) in the young subjects, but not in the elderly subjects. (3) The CoT was significantly greater in elderly subjects than young subjects at any speeds. (4) The slope of $\dot{V}$_E_–$\dot{V}$CO_2_ linkage was steeper in the elderly subjects compared to the young subjects.

### Age-Related Increase in Energy Expenditure With Increased $\dot{V}$_E_

In support of our second hypothesis, the CoT values in the ELD group was significantly greater than those of CoT the YG at any speeds (*p* < 0.01, **Table 1**). These results are in accordance with some previous studies (Peterson and Martin, 2010; Hortobágyi et al., 2011; Ortega et al., 2014). The walking economy should be deteriorated when aging. Other investigations showed that the preferred walking speed is slowed by aging (Mademli and Arampatzis, 2014; Schrack et al., 2016). In other words, the CoT is inversely proportional to the slower preferred walking speed in the elderly.

Our present study found that different walking speeds from 3 to 5 km h^-1^ in the ELD and from 3 to 6 km h^-1^ in the YG still yielded greater ΔCoT values in the ELD compared to the YG (**Figure 1**). Figueiredo et al. (2013) showed that the age-related increase in the CoT was attributed to an increased ventilatory cost during walking in patients with chronic heart failure. In our recent observation, the energy cost of circulation accounted for 80% of cardiopulmonary work at rest in all three different F_I_O_2_ conditions (Horiuchi et al., 2017), indicating that relative contribution of the energy cost of ventilation was 20% of cardiopulmonary work. In the present study, there was a significant correlation between the ΔCoT decrease and the Δ$\dot{V}$_E_ decrease in the YG (*r* = 0.625, *p* < 0.01) and ELD (*r* = 0.515, *p* < 0.01). These results suggest that the CoT is partly dependent on the $\dot{V}$_E_ (**Figure 1**).

### Slower HR Kinetics With Advancing Age

HR kinetics were found to change little with age in a step work protocol (Babcock et al., 1994), whereas Cunningham et al. demonstrated that HR kinetics in elderly women were slowed during a sinusoidal work protocol (Cunningham et al., 1992). Chilibeck et al. (1996) observed that the time constant of the HR during both cycling and walking were markedly different between elderly and younger individuals. In light of these findings, it could be concluded that the different profiles of HR kinetics are due to the use of different work loading such as a step work pattern and a sinusoidal work pattern.

As mentioned before, the cardiac vagal tone was dramatically reduced (by >110 beats min^-1^) in the HR response during an incremental work test (Yamamoto et al., 1991). Sone et al. (1997) indicated that the increased HR below 100 beats min^-1^ would seem to reflect the weakening in the contribution of the parasympathetic system to HR regelation during sinusoidal cycling. Indeed, we observed that the average values of HR under the five different sinusoidal work-loads showed a narrow range (from 95 to 98 beats min^-1^) in the YG; thus, the relatively lower value of HR must have been associated with parasympathetic nerve activity and might have contributed to the faster HR kinetics in the YG. Nor did we find any significant correlation between the CVHR and the *Amp* ratio of HR only in the ELD (**Figure 6**), which indicates that parasympathetic nerve activity does not play a predominant role in the HR kinetic system during mild sinusoidal walking; rather, this system seems to be administered by the age-related reduction in parasympathetic outflow, because a significant correlation between the *Amp* ratio and the CVHR was observed only in the YG. Thus, our first hypothesis was supported.

Taken together, the results of this study showed that HR kinetics in response to mild sinusoidal walking are more closely related to advancing age than gas exchange kinetics. In addition, the CVHR and the *Amp* ratio of the subjects’ HR were not closely correlated only in the ELD (**Figure 6**). This observation is evidence that the HR kinetics in response to mild sinusoidal work is attributable to sympathetic neural control in the heart. These results clearly demonstrate that the HR kinetics during walking was highly sensitive to aging.

### Ventilatory Equivalent CO_2_ ($\dot{V}$_E_/$\dot{V}$CO_2_) With Aging

As shown in **Figure 5B**, the $\dot{V}$_E_/$\dot{V}$CO_2_ slope was greater in the ELD (0.0258) compared to that (0.0132) in the YG. A significantly greater $\dot{V}$_E_/$\dot{V}$CO_2_ slope of ∼0.030 was observed in the ELD, which was to compensate for their increased physiological dead space, compared with that in the YG (Brischetto et al., 1984; Inbar et al., 1994). Moreover, there is much interesting that greater $\dot{V}$_E_/$\dot{V}$CO_2_ slope is associated with an augmented chemoreceptor sensitivity in patients with chronic heart failure (Ponikowski et al., 2001a,b). Thus, third hypothesis could be supported.

A greater correlation between *Amp* of $\dot{V}$_E_ and $\dot{V}$CO_2_ was also found in the ELD (*r* = 0.856, *p* < 0.01) compared to the YG (*r* = 0.703, *p* < 0.01), which indicates that age-induced ventilatory fluctuation is more closely linked to fluctuations in metabolism through CO_2_ production. Similarly, the ventilatory response to hypercapnia was significantly lower in elderly individuals compared to young individuals (Brischetto et al., 1984). We could not directly observe the CO_2_ ventilatory responsiveness, but there is still a possibility that aging might have already lowered the CO_2_ ventilatory responsiveness of our ELD subjects. Thus, our results regarding the $\dot{V}$_E_–$\dot{V}$CO_2_ relationship do not support a scenario for the ventilatory response during exercise related to CO_2_ ventilatory responsiveness.

### Methodological Considerations

Although the ELD have not performed regular sports activity for the past 3 years, physical activity in elderly individuals could not be measured, thus, we could not control the influence of physical activity in response to cardiorespiratory kinetics during walking. Moreover, walking speeds were fixed at a maximum speed of 6 km h^-1^ and minimum speed of 3 km h^-1^ in young subjects at a maximum speed of 5 km h^-1^ and minimum speed of 3 km h^-1^ in elderly subjects. Since the elderly walked on the treadmill at 6 km h^-1^ difficulty, we selected maximum speed of 5 km h^-1^ in order to adjust the equivalent metabolic demand in both groups. For example, HR max is estimated 193 beats min^-1^ in the YG and 161 beats min^-1^ in the ELD using *eq*. HR max = 208 – (0.7⋅Age) (Tanaka et al., 2001), resting HR in this study were averaged 78 beats min^-1^ in the ELD and 61 beats min^-1^ in the YG, respectively. HR reserve at 100 beats min^-1^ was estimated at 29.5% in the YG and 26.5% in the ELD, indicated that HR reserve was similar between the YG and ELD. Therefore, the relative intensity (HR reserve) and absolute energy demand (*Mx*) in the ELD during sinusoidal walking were similar those in the YG, these phenomenon also demonstrate the differential responses of the cardiac and metabolic variables during walking.

## Conclusion

We found that the *Amp* ratio of HR was significantly related to the CVHR in the young subjects, but not in the elderly subjects. This may indicate that the HR kinetics during sinusoidal walking may not be attributable to parasympathetic nervous activity into the heart in the elderly. The CoT of the ELD was significantly greater than that of the YG. Moreover, the slope of $\dot{V}$_E_–$\dot{V}$CO_2_ linkage was steeper in the elderly subjects compared to the young subjects. From the viewpoint of clinical assessments, $\dot{V}$_E_–$\dot{V}$CO_2_ linkage can produce a valuable information even for healthy elderly because higher $\dot{V}$_E_/$\dot{V}$CO_2_ slope is associated with an augmented chemoreceptor sensitivity in patients with chronic heart failure (Ponikowski et al., 2001a,b).

## Author Contributions

NE and YF conceived and designed the study. TH, AA-A-D, and YM contributed to the collection of data. DA, MH, NE, and YF interpreted the data. All authors reviewed and approved the final manuscript written by NE, TH, and YF.

## Conflict of Interest Statement

The authors declare that the research was conducted in the absence of any commercial or financial relationships that could be construed as a potential conflict of interest.
